# Disaster Preparedness in Hospitals

**DOI:** 10.7759/cureus.50073

**Published:** 2023-12-06

**Authors:** Janhavi Khirekar, Ankit Badge, Gulshan R Bandre, Shivani Shahu

**Affiliations:** 1 Hospital Administration, School of Allied Health Sciences, Datta Meghe Institute of Higher Education and Research (Deemed to Be University), Nagpur, IND; 2 Microbiology, Datta Meghe Medical College, Datta Meghe Institute of Higher Education and Research (Deemed to Be University), Nagpur, IND; 3 Microbiology, Jawaharlal Nehru Medical College, Datta Meghe Institute of Higher Education and Research (Deemed to Be University), Wardha, IND

**Keywords:** public awareness, risk assessment, emergency management, safety plan, disaster preparedness

## Abstract

Disaster preparedness in hospitals is a critical global concern that involves proactive measures to mitigate the impact of natural or artificial disasters. The review emphasizes the role of organizations such as India's National Disaster Management Authority in the development of response strategies. Hospitals face challenges in protecting facilities and healthcare workers during disasters, highlighting the need for effective training, equipment, and communication access. Differentiating disasters into natural, technological, and artificial types showcases the varied challenges each presents. Key challenges include resource allocation, interoperability of the communication system, evacuation strategies, and ethical considerations. Essential strategies include risk assessment, staff training, communication, and collaboration with external partners. Hospital disaster preparedness requires a comprehensive approach that involves strategies, training, and community participation to ensure safety during emergencies.

## Introduction and background

Disaster is a rapid natural situation with enough significance to require external support. Disaster preparedness refers to proactive measures and plans put in place by individuals, communities, and organizations to minimize the impact of natural or artificial disasters [1,2]. The National Disaster Management Authority is India's apex body responsible for disaster management. It involves developing emergency response strategies, creating disaster management systems, conducting drills and simulations, stockpiling essential supplies, and educating the public about potential hazards and protective action [3]. Disaster preparedness for hospitals is a global issue. Disaster damage to healthcare facilities is a human tragedy that causes great financial losses, obliterates development goals, and erodes communal trust. Making hospitals and other healthcare facilities disaster-proof is socially and morally cost-effective [4,5]. The healthcare sector is tasked with protecting people's health and safety as a priority. One of the most pressing health problems that is developing and that must be addressed is the treatment of diseases caused by biological events [6]. The government has changed disaster management structures, methods, and policies over time. In conjunction with developments in the disaster risk management system, the health system has evolved, moving from a focus on primary healthcare to a traditional curative strategy [7]. The global coronavirus disease 2019 (COVID-19) pandemic has caused a health and economic crisis, underscoring the importance of preparedness for disasters and resilience to hazards. It has exposed vulnerabilities in healthcare systems, supply chains, and social support structures [8]. The objective of this review is to discuss the importance of disaster management and the role of healthcare preparedness in disaster management.

## Review

Importance of disaster preparedness in hospital

In an emergency, hospitals must be equipped to handle patient overflow. To effectively assess, treat, and manage patients with various degrees of disease and injury, they must have the right strategies, processes, and tools. The possibility of further harm and improving the delivery of healthcare, adequate disaster preparation ensures the safety of patients [9]. Doctors, nurses, and support workers in hospitals are essential resources in the event of a disaster. For medical facilities to be maintained and responses to be effective, people's security and well-being are essential. To ensure their safety and ability to provide care, healthcare workers must be trained in emergency procedures, given the protective equipment they need, and given access to communication networks. This is known as disaster preparedness [10]. Natural disasters can burden healthcare resources such as medical supplies, appliances, and employees. Hospitals with comprehensive disaster preparedness plans can better manage and allocate their resources, provided they are used effectively and efficiently. This involves keeping reserves of the necessary medicines, medical equipment, and other supplies and establishing procedures to monitor and refill them during and after a crisis [11]. Collaboration and cooperation with numerous stakeholders, including regional emergency response agencies, public health departments, and neighborhood organizations, are essential for effective hospital disaster preparedness. Hospitals can contribute to a coordinated response system, pool resources, exchange information, and streamline communication channels by actively participating in emergency preparedness initiatives. This will enable a coordinated and successful response to catastrophes [12]. The key aspects of hospital disaster preparation are as follows (Table 1).

**Table 1:** Aspects of hospital disaster preparedness

Aspects	Description
Strategies and processes	Hospitals need effective strategies and processes to assess, treat, and manage patients during emergencies [9].
Tools	Equipping hospitals with appropriate tools is crucial to managing patient overflow and various degrees of infection [9].
Safety of patients	Disaster preparation ensures patient safety, minimizes further harm, and improves healthcare delivery [10].
Healthcare workers	Training, protective equipment, and access to communication networks are vital for their safety [10].
Resource management	Comprehensive plans help hospitals manage and allocate resources, such as medical supplies and equipment, effectively [11].
Collaboration	Working with emergency agencies and stakeholders is crucial to a coordinated response and the aggregation of resources [12].

Types of hospital disasters

Disasters of different types can disrupt hospital operations and represent considerable obstacles to patient care. Hospitals often have emergency management processes to deal with these various types of disasters and maintain the safety and well-being of clients, employees, and the community [13].

Natural Disasters

Natural disasters are extraordinary events or events caused by natural forces and can potentially cause significant damage, destruction, and even death. These occurrences are often beyond human control and can have terrible repercussions for the affected area and its residents. Natural disasters, including earthquakes, hurricanes, floods, wildfires, tornadoes, and severe storms, can impact hospitals. These events can cause evacuation of the patient, infrastructural damage, and problems with the water and electrical systems. Without functioning hospitals, the affected area may struggle to provide medical care to those in need, leading to further loss of life [14]. Additionally, the destruction of hospitals can also disrupt the training and education of healthcare professionals, making it even more difficult to rebuild and recover after a natural disaster. It is crucial that communities have emergency plans in place to ensure the resilience and preparedness of their health systems in the face of these devastating events [15].

Technological Disasters

A technological disaster in a hospital is when there is a substantial breakdown or issue with the hospital's technology infrastructure, which affects patient care, raises safety concerns, and could even endanger people. When a technical disaster occurs in a hospital, quick action is required to mitigate the effects and resume regular operations [16]. Strategy often includes setting up backup systems, hiring information technology specialists, alerting appropriate authorities, ensuring patient safety, and implementing contingency plans to deal with the problem. Communication with patients and their families is crucial during a technical disaster. Hospital staff must inform patients about the situation and ensure that their care is not compromised. This may involve providing alternative communication methods, such as phone or email, to keep patients updated on their treatment plans and any changes in their scheduled appointments [17].

Artificial Disaster

An incident caused by human behavior or carelessness that is detrimental to hospital operations, patients, personnel, or infrastructure is an artificial disaster. To prevent or reduce the effects of manufactured disasters, hospitals must have robust safety procedures, regular staff training, and effective risk management plans. These measures help create a culture of safety within the hospital, ensuring that all personnel know potential hazards and how to respond in emergencies. Maintaining current equipment and technology is crucial to preventing artificial disasters, as faulty machinery or outdated systems can cause accidents or malfunctions. By prioritizing safety and implementing these preventive measures, hospitals can minimize the impact of artificial disasters and provide a safe environment for patients and staff [18].

Role of healthcare professionals in disaster preparedness

Healthcare personnel are essential for hospital disaster preparedness. They can respond to emergencies and provide vital medical care during a crisis due to their knowledge and training. By ensuring that hospitals are equipped, trained, and prepared to provide quality medical care during disasters, healthcare workers play a crucial role in disaster preparedness [19].

Leadership and Decision Making

Leadership and decision-making are two connected concepts crucial for the growth of individuals, teams, and communities. Leadership is the capacity to inspire, motivate, and influence people to achieve a common goal. Strong decision-making skills are necessary for effective leadership because leaders often find themselves in situations where they must make important decisions that influence their team, organization, or community. Making decisions and acting as a leader go hand in hand. Influential leaders show that they have good decision-making abilities by having a clear vision, acquiring pertinent data, taking stakeholders into account, assessing risks, encouraging teamwork, and acting ethically. Additionally, the acquisition of pertinent data allows leaders to make informed decisions, ensuring that they consider all available information before taking action [20,21].

*Emergency* *Medical Services *

The term Emergency Medical Services (EMS) refers to a network of medical facilities set up to offer rapid medical aid in the case of emergencies. These facilities include ambulance services, hospitals, and trained medical personnel trained to handle critical situations. EMS saves lives by providing immediate medical attention, stabilizing patients, and transporting them to appropriate healthcare facilities. The efficiency and effectiveness of emergency services are essential to ensure timely intervention and increase survival chances for those in need [22]. EMS is essential to get patients to trauma centers for additional testing and care. To facilitate a smooth transition to treatment, emergency department personnel work closely with emergency service workers. Although emergency medical services systems may differ between nations and areas, their essential objective of providing prompt and efficient medical care in emergencies remains the same. These treatments are essential to save lives, lessen the effects of injuries, and improve patient outcomes [23].

Triage and Patient Management

Triage is the initial evaluation of patients to decide which ones need to be seen first by a doctor. To stop further deterioration or loss of life, it seeks to identify patients who need immediate or urgent care. Triage is often carried out by qualified healthcare professionals who adhere to established norms and guidelines, such as nurses or emergency medical personnel [24]. Patient management entails providing the appropriate treatment according to each patient's priority once they have been triaged and classified. This involves allocating resources and personnel to ensure that patients receive timely and appropriate care. Patient management also includes monitoring and reevaluating patients as their condition may change. It is crucial for healthcare professionals to communicate effectively and collaborate with each other to ensure the smooth flow of patient care and prioritize those in critical condition. The ultimate goal of patient management is to ensure optimal outcomes for all patients and provide them with the best possible care [25].

Challenges in hospital disaster preparedness

Planning, organizing, and implementing ideas are all parts of hospital disaster preparation that involve managing many crises or disasters. However, the unique difficulties faced by each hospital can differ based on elements, including location, size, and resources [26].

Resource Allocation

Resource allocation is the efficient allocation of available resources to various tasks, projects, or activities within a system or organization. In addition to intangible resources such as time, talent, and knowledge, resources can also comprise actual assets such as cash, machinery, supplies, and staff. Achieving targeted results while minimizing expenses and increasing productivity requires an effective allocation of resources. It involves determining how many resources are needed for different projects or activities and determining their capacity and availability [27].

Interoperability and Communication Systems

The capacity of various systems or components to cooperate and exchange information is called interoperability. Interoperability in the context of communication systems refers to the ability of various devices, networks, or protocols to communicate and share data. Interoperability is crucial in the interconnected world of various technologies and platforms. Communication systems must be interoperable to facilitate effective and seamless communication between various organizations. The interoperability capacity to increase connection, increase efficiency, and stimulate innovation is crucial for communication systems. Without interoperability, data silos would hinder productivity and hinder the development of advanced solutions based on the integration of multiple technologies [28].

Evacuation and Sheltering

Two crucial components of emergency readiness and response in disasters, natural disasters, or other dangerous events are evacuation and sheltering. Evacuation is the act of removing individuals to a safer place from a potentially hazardous or high-risk environment. The objectives are to protect people from imminent danger, reduce the possibility of harm or death, and maintain their safety during the crisis [29]. When evacuation is not an option or is not advised, providing a safe and secure location for people to stay during an emergency or disaster is known as sheltering. The quick start of an emergency or the lack of adjacent safe evacuation routes are only two examples of situations in which sheltering may be essential [30].

Ethical Considerations

In many different industries, including technology, healthcare, business, and research, ethical concerns are a crucial component of behavior and decision-making. Ethical concerns encompass a wide range of issues, such as privacy, data security, fair labor practices, and environmental sustainability. Adherence to high ethical standards not only fosters trust and credibility but also ensures long-term success and positive social impact [31]. It involves evaluating how decisions could affect numerous stakeholders, including people, communities, society, and the environment. Ethical issues are crucial in many different industries, including business, technology, research, and medicine. To ensure ethical and responsible behavior, it is crucial to critically assess and manage ethical challenges [32]. Challenges in hospital disaster preparedness are as follows (Table 2).

**Table 2:** Challenges in hospital disaster preparedness

Topic	Description
Resource allocation	Efficient management of tangible and intangible tasks and projects [27].
Interoperability and communication systems	Systems' ability to exchange data and work together. Crucial for seamless communication among various devices and networks, fostering efficiency and innovation [28].
Evacuation and sheltering	Vital in emergencies. Evacuation: moving people from danger to safety. Sheltering: providing safe spaces when evacuation is not possible. Objectives: protect lives and ensure safety [29,30].
Ethical considerations	Essential in various industries. Involves evaluating impacts on stakeholders (people, society, environment) to ensure responsible decision making [31].

The essential strategies for disaster preparedness in hospital

During an emergency, disaster preparedness in hospitals is critical to ensuring the safety and well-being of patients, employees, and the community. Here are some critical hospital catastrophe preparation techniques [33]. Create an emergency operations plan (EOP) for hospitals. Hospitals should have an EOP that includes roles, duties, and processes for many disasters. The plan should be regularly updated, analyzed, and practiced through training and experiments [34]. The risks of potential dangers and risks specific to the location and facility should be assessed. Disasters caused by nature, technical hazards, and artificial threats are considered [35]. Set up the command and control. Develop an incident command system and a transparent chain of command during an emergency. This ensures that hospital personnel, emergency responders, and related agencies collaborate effectively to coordinate, communicate, and make decisions [36,37]. The maintenance of critical infrastructure and critical services systems, such as power supplies, water, sanitation, and medical gas systems, should be identified and maintained by hospitals during disasters. Create backup strategies for different resources or recovery systems [38]. Collaboration with external partners is essential. Form alliances and interact with regional emergency management agencies, health care departments, and other health care facilities. This promotes the exchange of resources, mutual aid, and coordinated response actions [39,40]. Perform hazard-specific planning, which makes disaster contingency strategies specific to hospital areas, such as hurricanes, volcanic eruptions, or emergencies. Consider the unique needs of people with special needs, such as children and older people. Incorporate evacuation plans that consider mobility limitations and ensure that adequate resources are available to meet their medical needs [41]. Additionally, establish communication channels with local emergency management agencies to stay informed of potential hazards and collaborate on response efforts. By implementing these hazard-specific planning measures, hospitals can better protect their patients and staff during times of crisis [42,43]. Evaluation and improvement are done by utilizing post-incident discussions, after-action reports, and input from workers and supporters to continuously assess the success of disaster preparedness initiatives. Update and improve hospital emergency preparedness strategies using this information. The hospital can take advantage of valuable on-the-ground insights and expertise by actively involving workers and supporters in the evaluation process. This collaborative approach helps improve hospital emergency preparedness strategies, making them more effective and adaptable to future potential disasters [44,45]. Strategies used in hospital disaster preparedness are as follows (Table 3).

**Table 3:** Strategies used in hospital disaster preparedness EMS: Emergency medical services

Strategies	Description
Risk assessment and planning	Planning and risk assessment are essential for efficient project leadership and decision-making processes. Identifying potential hazards, assessing their potential effects, and estimating their propensity to occur are all parts of the risk assessment process. On the other hand, planning involves creating plans of action and strategies to minimize or handle those risks that have been recognized [46,47].
Emergency response and triage	Critical elements of EMS and disaster management include emergency response and assessment. The emergency response describes the quick decisions made by first responders, such as medical professionals, firefighters, and police, to provide help and support in dire circumstances. The process of grouping patients according to the urgency of their condition and the availability of resources is called triage [48].
Staff training and education	The professional development and advancement of employees are highly dependent on education and training. Companies can develop their employees' abilities, expertise, and knowledge by offering learning and educational opportunities, resulting in improved performance, increased production, and overall organizational success [49].
Communication and information management	Each organization must have effective communication and information management. Collaboration, decision-making, and problem-solving are more accessible when information is effectively shared between people or groups. Information management entails putting information in an effective, safe, and accessible format for the appropriate stakeholders while organizing, storing, retrieving, and disseminating it [50].
Resource management	The process of organizing, allocating, and managing different resources within a company to ensure efficient and effective utilization of those resources. In addition to intangible resources such as staffing, time, and skills, resources can also comprise tangible assets such as money, materials, equipment, and infrastructure [17].
Mitigation resources	Disaster mitigation measures are acts and methods implemented to limit the impact of prospective disasters and minimize the harm they can cause. A multifaceted approach that incorporates these strategies and community participation and cooperation is critical to successful disaster mitigation [51].

## Conclusions

Disaster preparedness in hospitals is critical to effectively managing crises. It involves proactive measures such as efficient strategies, trained staff, and appropriate tools to ensure patient safety and manage resources during emergencies. The challenges include resource allocation, interoperability of the communication system, evacuation, and ethical considerations. Natural, technological, and artificial disasters each require specific strategies, emphasizing the need for emergency plans and safety measures. The role is crucial, relieving leadership, decision-making, and EMS to prioritize patient care. Strategies involve risk assessment, training, communication, and resource management, to mitigate the impact and ensure rapid response. Collaboration and community participation are key to successful disaster readiness, allowing hospitals to navigate various crises efficiently.
